# A multi-centre prospective evaluation of THEIA™ to detect diabetic retinopathy (DR) and diabetic macular oedema (DMO) in the New Zealand screening program

**DOI:** 10.1038/s41433-022-02217-w

**Published:** 2022-09-03

**Authors:** Ehsan Vaghefi, Song Yang, Li Xie, David Han, Aaron Yap, Ole Schmeidel, John Marshall, David Squirrell

**Affiliations:** 1 Toku Eyes®, Auckland, New Zealand; 2 School of Optometry and Vision Science, The University of Auckland, Auckland, New Zealand; 3 Department of Ophthalmology, The University of Auckland, Auckland, New Zealand; 4 Department of Diabetes, Auckland District Health Board, Auckland, New Zealand; 5 Institute of Ophthalmology, University College of London, London, UK

**Keywords:** Outcomes research, Technology

## Abstract

**Purpose:**

To validate the potential application of THEIA™ as clinical decision making assistant in a national screening program.

**Methods:**

A total of 900 patients were recruited from either an urban large eye hospital, or a semi-rural optometrist led screening provider, as they were attending their appointment as part of New Zealand Diabetic Eye Screening Programme. The de-identified images were independently graded by three senior specialists, and final results were aggregated using New Zealand grading scheme, which was then converted to referable/non-referable and Healthy/mild/more than mild/sight threatening categories.

**Results:**

THEIA™ managed to grade all images obtained during the study. Comparing the adjudicated images from the specialist grading team, “ground truth”, with the grading by the AI platform in detecting “sight threatening” disease, at the patient level THEIA™ achieved 100% imageability, 100% [98.49–100.00%] sensitivity and [97.02–99.16%] specificity, and negative predictive value of 100%. In other words, THEIA™ did not miss any patients with “more than mild” or “sight threatening” disease. The level of agreement between the clinicians and the aggregated results was (*k* value: 0.9881, 0.9557, and 0.9175), and the level of agreement between THEIA™ and the aggregated labels was (*k* value: 0.9515).

**Conclusion:**

This multi-centre prospective trial showed that THEIA™ did not miss referable disease when screening for diabetic retinopathy and maculopathy. It also had a very high level of granularity in reporting the disease level. As THEIA™ has been tested on a variety of cameras, operating in a range of clinics (rural/urban, ophthalmologist-led\optometrist-led), we believe that it will be a suitable addition to a public diabetic screening program.

## Introduction

Implementation of artificial intelligence (AI) in medicine and particularly in ophthalmology has a long history, but also accelerating rapidly in the past few years [1–4]. So far, the most promising application of AI in ophthalmology is as a screening tool for Diabetic Retinopathy (DR) [5–9].

It is now well accepted that a comprehensive DR screening program can reduce the burden of diabetes related vision loss [4, 10, 11]. However, delivering large community-based programs can be a major challenge even in developed countries, such as including New Zealand which has both a high prevalence of diabetes [12] and a significant proportion of the population not being screened regularly [13]. AI based algorithms, that can reliably detect DR in retinal images and provide instantaneous reporting with high diagnostic accuracy, could significantly improve the earlier detection of DR. In addition, by enabling specialist-level diagnostics to be provided to multiple peripheral sites simultaneously these algorithms also have the potential to significantly increase access to, and lower the cost of, screening for DR [14, 15].

In recent years there have been significant advances in development of AI algorithms to assist with diabetic eye screening programs [1]. While the accuracy of AI-based models for detecting DR have been demonstrated in many previous studies [5–9], most have failed to perform in the “real world” setting [16]. It has been shown that most research AIs for detection of retinopathy are not generalizable, as training datasets used are not representative of the wider society, obtained from relatively homogenous populations, limited in numbers or highly curated by clinicians, contain just one image per eye, and very limited grade granularity (i.e. binary outcome for referable disease) [17].

Toku Eyes^®^ in partnership with the Auckland and Counties Manukau District Health Boards (DHB) in New Zealand developed THEIA™, a AI DR Screening tool that is: trained and tested locally, is clinic/clinician/camera agnostic, gender/age/ethnicity unbiased, and provides retinopathy and maculopathy grading to the New Zealand Ministry of Health requirements [18]. The preliminary results of the first iteration of THEIA™, a trained and tested on a large dataset that represents 25% of New Zealanders living with diabetes, demonstrated a sensitivity of [94–95%] and specificity of [61–63%] for sight threatening DR [5]. The algorithm has subsequently been improved and optimized by a process of continuous retraining and testing. In this paper, the results of the latest iteration of THEIA™ tested in a prospective multi-centre prospective trial, where the patients were recruited from two New Zealand National Diabetic Screening programs; a regional community Optometrist based provider and a Central Auckland DHB provider, are presented. Each of these two programs serve different communities employing a variety of cameras in their screening service. The aim of this study was to establish the efficacy of THEIA™, regardless of the type of fundus camera being used for, or location of the screening centre [5]. In this paper, the results of a bespoke AI are presented, one that was developed to provide primary screening of diabetic retinopathy to augment the existing DR screening program in New Zealand; one that both accurately represents the real-world DR screening environment and is representative of the patients it is designed to serve.

## Methods

### Study population

This was a prospective study, where participants were recruited from two separate clinics that are participating in the New Zealand Diabetic Screening program. One is a large urban tertiary DHB clinic, the other clinic was located in a provincial optometric practice. The central DHB service used a variety of 45 degree non mydriatic cameras at its different sites; Canon DGi (2 units), Canon CR2 (2 units) and Canon CR2 + AF (2 units), while the optometric led centre was using an iCare EIDON camera. The study protocol was approved by the Health and Disability Ethics committee at New Zealand Health and Disability Ethics Committee (20/STH/178) and Counties Manukau Health (CMH-947). The trial is registered on the ANZCTR, Registration number ACTRN12620000488909 and has been issued with the Universal Trial Number (UTN) U1111-1249-7630.

Consecutive patients attending for a publicly funded retinal screening (within the eye hospital or optometric setting) between January 2021 and April 2021, over the age of 21, were invited to participate. The only exclusion were patients who were unable to give their consent. To ensure that there was a sufficient number of patients with diseased images, the study remained open until the desired number of patients with disease had been recruited.

The process of DR screening in New Zealand has been outlined previously [5], but in brief all participants with Type 2 diabetes mellitus (T2DM) were photographed twice in each eye, i.e. one macula-centred and one disk-centred image, and all patients with Type 1 diabetes mellitus (T1DM) were imaged four times in each eye, with an addition two images taken one below the disc and one above the disc. All patients are initially photographed through undilated pupils, pupil dilation being used if the image that was subsequently acquired was deemed by the photographer to be inadequate. At the conclusion of data collection, the images were de-identified and assigned a unique patient ID by an independent technician.

The de-identified images were then passed on to three independent specialists who oversee the DR grading teams at each of the 3 metro Auckland DHBs screening programs. Each graded the entire dataset independently, according to New Zealand Ministry of Health standards [18]. The grading happened simultaneously, and the individual graders were masked to the grades issued by the other two graders. Where there was a discrepancy in the grades issued by the three independent graders a fourth independent, senior retinal specialist was used to adjudicate the outcome. An adjudicated master ‘ground truth’ list was thus created by aggregating the three independent reports. The level of agreement between graders and the adjudicated ground truth was assessed using both a kappa statistic and percentage agreement. This adjudicated data set formed the “ground truth” against which THEIA™ was subsequently independent to the human grading pathway, the de-identified colour images were analysed by the THEIA™ AI platform, by means of uploading images to its dedicated Amazon Web Services (AWS) portal. THEIA™ has been described in detail previously but in brief comprises a Quality assurance AI installed on the capture station and a grading AI that is hosted in the Cloud [5, 6]. The QA function is designed to assess whether the image captured is of acceptable Quality for the suite of grading AI’s to read. If the image is of acceptable quality the user “accepts” the image which is, then sent to the grading AI’s for analysis. If the image is not of adequate quality the user is invited to take further images to secure images that are of sufficient quality. If this is not possible the user is then presented with the choice of abandoning digital imaging and sending the patient for slit lamp review or overriding the inbuilt image QA alert and sending the images for grading regardless.

The THEIA™-generated grades were then compared with the ground truth by way of confusion matrices. Using grades derived from the New Zealand grading system [19], the efficacy of THEIA™ was assessed at the patient-level, using both a simplified binary referrable/non-referable grading scheme and the more global (aggregated) grading scheme of Healthy, mild, more-than-mild (mtmDR) and Sight threatening DR (Table 1). Where a discrepancy existed between the results issued by THEIA™ and the adjudicated ground truth, the images were reassessed by the group who, being masked to the origin of the results and the results issued by THEIA™, were asked to either agree with one of two the outcomes presented. Although the performance of THEIA™ could be reported at either the image, eye or patient level, as it has been designed primarily for use as a Clinician support tool to perform primary grading within a Diabetic eye screening program (DRS) we have chosen to lead with the PATIENT level binary Non referrable/ Referrable data. For sake of transparency all Patient and Eye level data will be presented.

**Table 1:** Aggregation of New Zealand diabetic screening standard grades to the international (None Detected, mild, more-than-mild, sight threatening) disease gradings.

	Retinopathy	Maculopathy	Retinopathy & Maculopathy
None Detected	R0	M0	R & M = 0
Mild	R1, R2	M1, M2	R & M < 3
mtmDR	R3	M3	R or M = 3
Sight threatening	R4, R5	M4, M5	R or M > 3, R & M = 3

None Detected and Mild NPDR = Non referrable disease.

mtmDR and Sight threatening DR = Referable disease.

Disease classification further reduced to a binary referable/non referable classification as illustrated.

### Statistical power calculation

The primary study outcome was the sensitivity and specificity performance of the AI to detect referrable retinopathy. Study success was thus pre-defined as both sensitivity and specificity of the AI system in the New Zealand population. The hypotheses of interest are H0: *p* < p0 vs: HA: *p* > p0 where p is the sensitivity or specificity of the AI system and p0 = 75% for the sensitivity endpoint and p0 = 77.5% for the specificity endpoint under the null hypotheses.

The alternative hypotheses were 85% for sensitivity and 82.5% for specificity, reflecting anticipated enrolment numbers and pre-specified service requirements. One-sided testing was further prespecified for both sensitivity and specificity; a one-sided 2.5% Type I error was used resulting in a one-sided 97.5% rejection rule per hypothesis. To preserve Type I error, study success was defined as requiring both null hypotheses to be rejected at the end of the study.

Sample sizes for these hypotheses were calculated for at least 85% power and one-sided 2.5% Type 1 error. This indicated that we required a minimum of 840 participants, at least 149 of whom had referable DR or DMO.

## Results

**Fig. 1:**
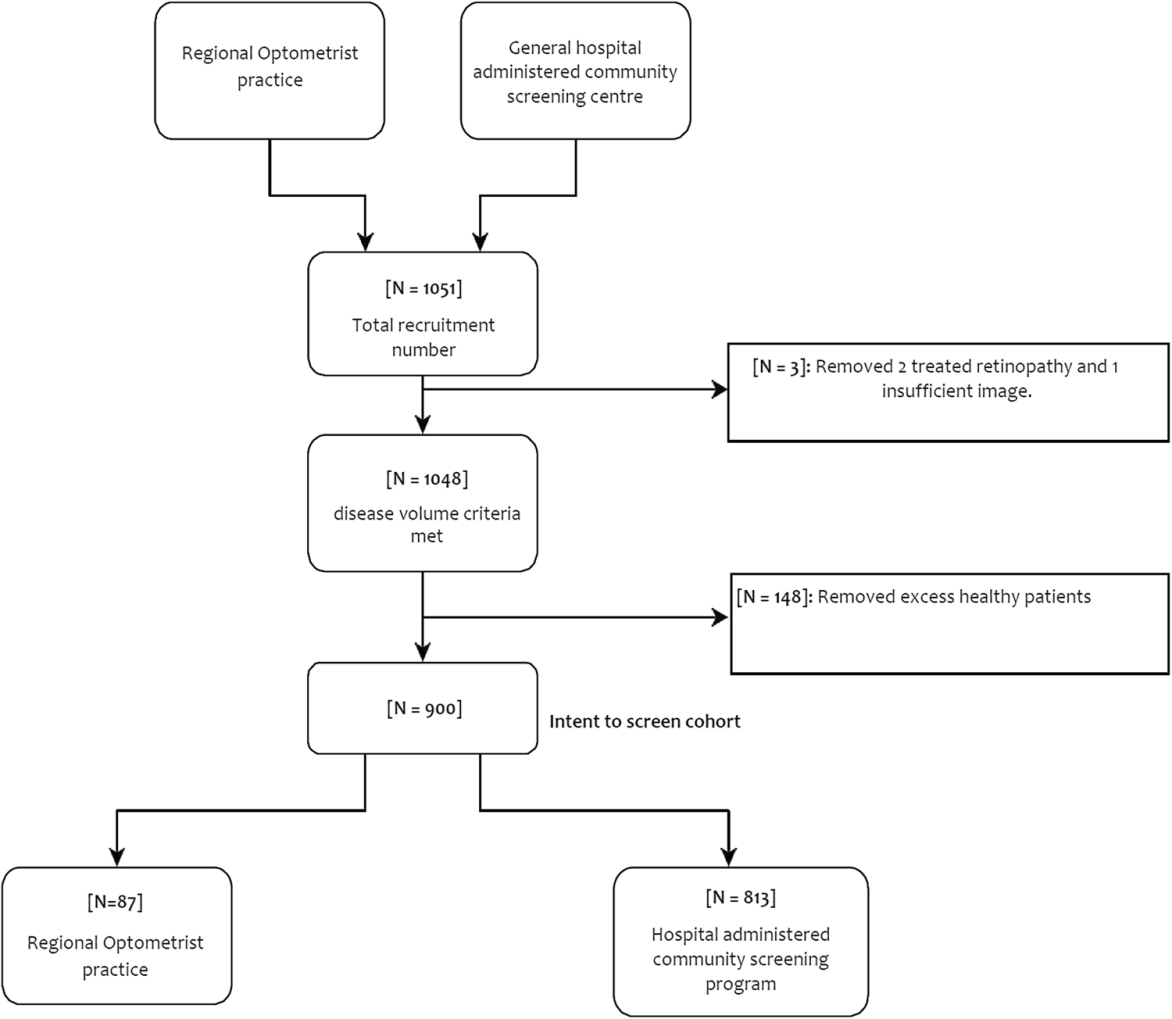
Study recruitment flow. The breakdown of participants analysed for the THEIA V1 Clinical Study, from enrolment to analysis.

At the time of the study, and to address the large backlog that had resulted from the COVID lockdowns in New Zealand, the diabetic eye screening programs were prioritizing high risk patients with historically suboptimal diabetes control or established retinopathy. It was anticipated that this would result in recruiting a higher number of patients with disease than would usually be the case. Images were read sequentially but because there was both a time lag between the dates the results were issued and the date the patient was recruited and multiple sites were involved, more patients were recruited than the power calculation required (total recruitment 1048). 246 individuals recruited into the study had disease that was deemed to represent referrable disease. Of these 2 had previously treated proliferative DR with extensive pan retinal photocoagulation and in 1 there was an insufficient set of images to be accurately graded. These three participants were therefore excluded from the final analysis. 243 patients with referrable disease were therefore enrolled into this study. The remaining patients (804) had none or minimal disease. As the study had over recruited, to minimize the burden on the grading team, the dataset of patients with none or minimal disease was reduced by random selection to 657 patients to make a total of 900 patients. This curated image dataset was then presented to the studies grading team. The final calculations were therefore based on a total of 900 patients (Fig. 1). THEIA™ managed to grade all the images that were acquired during this study, regardless of the site, camera, or the operator.

### Results Binary Non referrable/referrable classification

The Patient-level results, using the simplified 2 class classification Non referrable (R0-R2, M0-M2) vs Referrable (R3-R5, M4-M5), and using the worst R or M outcome in either eye is shown in Table 2. THEIA™ achieved a 100% sensitivity and 98.18% specificity, with the overall accuracy of 98%. Using the binary Non-referrable/referrable classification, the level of agreement between the three individual graders and the gold standard and THEIA™ and the gold standard was extremely high (*k* values: 0.98, 0.96, and 0.92 respectively for the 3 graders and THEIA *k* value: 0.95).

**Table 2:** Patient-level results using 2 class assessment scale referral/non-referrable for a large urban hospital, a provincial Optometrist led screening provider, and the combined data.

Overall	Accuracy	Confusion matrix	Specificity	Sensitivity
Central Auckland DHB	98.65%	[597 11] [0 205]	98.19%	100%
Optometrist LED Practice	99%	[49 0] [0 38]	100%	100%
Overall	98.78%	[646 11] [0 243]	98.33%	100%

The confusion matrix shows the predicted results in rows and ground truth in columns.

The Eye level results for the binary Non referrable/Referrable classification, broken down by the individual R and M grade results, are shown in Supplementary Tables 1–5. The sensitivity and specificity of detecting referrable retinopathy was 98.6% and 92.5% respectively. The sensitivity and specificity of detecting referrable maculopathy was 94.8% and 88.8% respectively.

### Results none/mild/more than mild/sight threatening

The Patient-level results; measuring THEIA™ performance against the gold standard with a granular 4 class classification; None (R0, M0), Mild (R1,2; M1,2), more than mild (R3, M3) and Sight threatening (R4,5; M4,5) using the worst R or M outcome in either eye, are illustrated in Table 3. These reveal that THEIA™ tended to marginally over grade disease, but it did not miss any patient with mtmDR disease or worse. THEIA™ issued a lower grade than gold standard in just 1 individual. They were issued with a mtmDR grade when the ground truth was considered to Sight threatening. The level of agreement between the three individual graders and the gold standard using the 4-class classification ranged from *k* value: 0.96–0.75. The corresponding level of agreement between THEIA™ and the gold standard was *k* 0.79.

**Table 3:** Patient-level THEIA™ confusion matrix using the (None Detected, Mild, mtmDR, Sight threatening) grading scheme for all participants.

	None Detected	Mild	MTMDR	Sight Threatening
None Detected	341	123	0	1
Mild	22	160	2	8
MTMDR	0	0	17	30
Sight threatening	0	0	1	195

The Eye level results for the 4-class classification, broken down by the individual R and M grade results are shown in Supplementary Tables 1–5. THEIA™ accurately graded the level of retinopathy in 1395/1713 (81.4%) of eyes and accurately graded the level of maculopathy in 1526/1702 (89.7%) of eyes.

THEIA™ demonstrated similar level of proficiency in identifying referable disease, in both the central DHB unit, and the Optometrist led practice (Supplementary Tables 6–8).

#### Audit of discordant grading

Using the 2 class classification system Referrable vs Non referrable, THEIA™ issued a different grade to the Gold standard adjudicated dataset in just 11 out of 900 patients. In all cases THEIA™ issued a grade that was higher than the gold standard (Table 2, Supplementary Fig. 1). Over grading of maculopathy resulted in 9 of the 11 cases where THEIA™ over graded compared to the gold standard. In 5 cases hard drusen were mistaken for exudate; Pachydrusen (2 cases) and thrombosed microaneurysms (2 cases) were responsible for the remainder. In 2 of the 11 cases where THEIA™ disagreed with the gold standard, THEIA™ issued an R3 grade instead of an R2 grade. In both cases the retinopathy was at the R2/R3 interface. The over-grading can be accounted for by the inbuilt “add up” function within THEIA™ which issued a patient level grade of R3 when the R grade classifier issued an R2 grade in both the disc and macular centred images. THEIA™ missed 4 cases (of 1713 eyes) where the eye level retinopathy was graded at mtmDR and 13 cases (of 1702 eyes) where the eye level maculopathy was graded as mtmDR (Supplementary Table 9).

#### Other pathologies detected

In addition to screening for DR, our grading team was asked to comment on other sight threatening pathologies. Two patients in the cohort had a hemorrhagic branch retinal occlusion and one patient had a central retinal vein occlusion. Although THEIA™ was not able to identify these diseases specifically, all three were identified by THEIA™ as having “referrable” disease. No other sight threatening pathology was identified in this cohort.

## Discussion

While there has been a flurry of research designed to create artificial intelligence tools for screening diabetic retinopathy (DR) or diabetic macular oedema (DMO), few algorithms have been tested prospectively in a real-world clinical environment [20–23]. This study, was designed to test the efficacy of our previously published algorithm (THEIA™) in a real-world prospective setting of two DR screening programs in New Zealand [5, 6] (Supplementary Table 10); one an urban DHB tertiary hospital screening centre the other a provincial Optometrist led screening centre. In this multi-centre prospective trial of 900 patients, at the patient level when a binary Non referrable/referrable classification was used THEIA™ achieved 100% imageability, 100% sensitivity, 98% specificity, with an overall accuracy of 98% for identifying referable disease when compared to an adjudicated gold standard. When a more granular classification of none. Mild, mtmDR and sight threatening disease was used THEIA™ missed no patient who had referrable disease as defined by either “mtmDR” or Sight threatening disease. The few inconsistent grades between THEIA™ and the adjudicated gold standard were largely a result of drusen; both small and hard drusen, and large pachydrusen [24], being mistaken for exudates. The gold standard adjudicated dataset was derived from grades issued by the senior lead grader in each of the three metro Auckland DHB screening programs. In keeping with their experience, the level of agreement between the individual graders and the adjudicated gold standard (*k* value: 0.92–0.98) when the data was aggregated into Referrable vs Non referrable disease, was excellent. Although no cases of referrable disease were missed, all three of the human graders marginally under-graded compared to the adjudicated gold standard. This result was not statistically significant. There was a comparable level of agreement between THEIA™ and the adjudicated gold standard (*k* value: 0.95). In contrast to the human graders, THEIA™ marginally over-graded the images, a result which is in keeping with a tool which is designed with a high sensitivity and thus designed not to miss disease. Overall, these results demonstrate that THEIA™ is both reliable and is as consistent as experienced specialist graders in diagnosing and detecting referrable diabetic retinopathy and maculopathy in the New Zealand (or similar) screening program.

As expected, the accuracy of the level of agreement, for both the human graders and THEIA™ was reduced when the more granular grading system; None Detected, Mild, mtmDR, Sight threatening, was employed. The apparent drop off in performance of both the human graders and THEIA™, (*k* value: human graders 0.96–0.75; THEIA 0.79), is a function of a number of compounding factors; the imposition of an ordinal scale onto a what is disease continuum leading to an increased probability of a mismatch at what is an artificial boundary of two disease states, and the increased numbers of boundaries that a more granular grading system imposes. To reduce the likelihood of missing disease THEIA™ has therefore been designed with an inbuilt bias to over grade in situations where the disease sits at the boundary threshold of two disease states. Reassuringly THEIA™ accurately predicted the correct grade of retinopathy in 82% cases of retinopathy and 89% cases of maculopathy when these two conditions were considered as different entities. When retinopathy and maculopathy grades were aggregated THEIA™ under graded sight threatening disease in just 7 cases, but in all cases THEIA™ still correctly identified them as “referrable” disease labelling them instead as “mtmDR”.

Compared to other algorithms which have been assessed prospectively in a real world setting [7–9, 25], THEIA™ performed very favourably. These results suggest that THEIA™ is capable of providing a very high granularity in the diagnosis of both retinopathy and maculopathy. Furthermore, unlike other clinically tested AIs [26–29], THEIA™ provides these disease grades based on all images acquired per screening visit with the whole process from image acquisition through to grading being completely automated. While there has been significant interest in developing diabetic retinopathy grading AIs [30], few have been trained to specifically grade diabetic maculopathy as a separate entity [26–29], this despite diabetic maculopathy being the commonest reason for Ophthalmology referral [31]. The performance of the retinopathy classifier was better than the maculopathy classifier, with most false positives being a result of over grading maculopathy. Grading maculopathy is more challenging than grading retinopathy [32]; firstly, exudate is used as a surrogate marker for oedema, and secondly there are several mimics of exudate, such as drusen, pachydrusen, focal ERM, that are difficult to discern without OCT. To address this issue screening programs in the UK and New Zealand have now started to incorporate OCT into their screening pathways. However, as most DR screening programs still operate an asynchronous model of care, and small hard drusen and focal reflective ERM are easily over looked at the time the patient attends for screening, these pathologies are often not identified until the retinal images are reviewed after the screening event. One advantage of using an AI such as THEIA™, which is capable of grading in real time, is that it facilitates the transition to synchronous models of care where patients can be issued their results at the point of care. An additional benefit of this model is that those patients who the AI identifies as having “suspected” maculopathy can be immediately imaged with OCT. This image could be read on the spot if telehealth support is available, or later if not. In either case there is no requirement for the patient to return as all the data required to grade their disease has been acquired.

THEIA™ has been designed primarily as a clinician assist primary triage tool. As such, it has been designed with an ultra-high sensitivity to ensure that sight threatening disease is not missed. In its previous configuration, THEIA™ achieved this at the expense of a modest specificity [5]. With a modification to the algorithm, the current version of THEIA™ preserved its ultra-high sensitivity while achieving a specificity higher than 95%. Whilst there was still a tendency for THEIA™ to over grade the issue of false positives is not overly troublesome because being a primary grading support tool it simply means that borderline images need to be read by a member of the grading team. The trade-off for the tendency to over grade is an ultra-high sensitivity and negative predictive value. Consequently, if THEIA™ grades an image as having no significant disease those responsible for the diabetic eye screening program can be confident that no significant disease has been missed. As most patients undergoing screening have minimal or no disease, we believe that the trade-off between “no disease missed” and a small number of false positives is reasonable. In this trial, four different camera types were used in multiple clinical settings; these included an iCare Eidon camera (confocal scanning laser ophthalmoscopy technology), and a variety of Canon cameras (conventional flash photography technology). THEIA™’s performance was unaffected by the camera type used or the shape and size of the image (Supplementary Tables 8 & 9). It also coped well with a number of artifacts on the real-world images including a central bright halo that was generated by one camera, and a random assortment of dot artefacts that appeared in a consistent location from another camera (Supplementary Fig. 2).

Whilst an accurate Algorithm is clearly important, there are several diverse issues that need to be addressed before AI can be safely incorporated into diabetic eye screening programs. These include but are not limited to equity, consent data privacy and stakeholder acceptance [17]. We have recently explored the attitude of patients undergoing retinal screening to the concept of using AI to read the retinal images acquired at the time of screening [33]. We found that although there is low awareness of clinical AI applications among our participants, most (78%) were receptive towards the implementation of AI in diabetic eye screening. In line with other similar surveys [34] there was a strong preference towards continual involvement of clinicians in the screening process and it is likely there will need to remain an option for those who prefer the service to be delivered manually [33]. These findings suggest that if clinical algorithm’s like THEIA™ are to be acceptable to stakeholders they will need to be deployed as primary grading support tools that augment the clinical teams at the point of care. Although a separate cost analysis of implementing THEIA™ was not part of this project, a team from Singapore have estimated that the adoption of a primary grading AI system, similar to THEIA™, would reduce the costs of delivering their existing DRS program by 20% [35].

The principal limitation of THEIA™ is that it cannot reliably identify other eye diseases that can present at the time of diabetic screening, such as glaucomatous optic neuropathy and age-related macular degeneration. Three patients in the current study had a significant retinal vein occlusion that was flagged up as significant retinopathy. It would also be reasonable to expect that haemorrhagic neovascular macular degeneration to be similarly identified. The Auckland DR screening program systematically records all other pathologies that are detected during routine screening. A recent analysis of this data revealed that only severe hypertensive retinopathy, retinal vein occlusion and macular degeneration are sufficiently important to justify systematic detection during routine diabetic eye screening [36]. Severe hypertensive retinopathy and many cases of advanced macular degeneration would already be picked up and flagged up by THEIA™ as mtmDR or Sight Threatening disease. Incorporating an AI classifier capable of detecting glaucoma suspects and intermediate and late AMD in addition to DR, would add further capability to THEIA™. Another potential limitation of AI is the ability of the algorithm to generalize to the population in which it is intended to be used. The population demographic that THEIA™ was trained has been described elsewhere [5]. Although the MoH in New Zealand does not keep a register of people living with diabetes, the Virtual Diabetes Register (VDR) [37] gives an estimate of the prevalence of diabetes in NZ, broken down by region and ethnicity. Comparison of the relative proportions of people living with diabetes in both the cohort who comprised the previously published retrospective study [5] and the current prospective study are similar to those reported in the VDR (Māori 18%, Pacific peoples 16%, Indian 6%, European/Asian/others 57%). We are therefore confident that our data is representative of the wider population of people living with diabetes in New Zealand and that the result of the current study therefore indicates that THEIA™ has successfully generalized to the population in New Zealand living with diabetes.

In conclusion, this multi-centre prospective trial demonstrates that THEIA™ is capable of detecting DR and DMO with a very high degree of accuracy, while providing a high level of granularity in grading. As such, and with appropriate oversight and audit, these results indicate that THEIA™ could be safely deployed within established diabetic screening programs to augment the expertise of the clinicians, increasing overall screening capacity while reducing costs per unit screen.

### Novelty statement

THEIA is proven to be the most accurate algorithm of its kind, through a double-blind prospective multi-centre trial. THIEA provides the highest level of disease diagnosis granularity, which is essential for early detection and timely intervention. THEIA provides an automated decision rule to ensure rapid, accurate classification of the large proportion of normal images from the few with abnormal features for prompt, accurate clinical grading, but not to replicate a screening program.

## Summary

### What was known before

- While there has been a flurry of research designed to create artificial intelligence tools for screening diabetic retinopathy (DR) or diabetic macular oedema (DMO), few algorithms have been tested prospectively in a real-world clinical environment. This study, was designed to test the efficacy of our previously published algorithm (THEIA™) in a real-world prospective setting of two DR screening programs in New Zealand.

### What this study adds

- This multi-centre prospective trial showed that THEIA™ did not miss referable disease when screening for diabetic retinopathy and maculopathy. It also had a very high level of granularity in reporting the disease level. As THEIA™ has been tested on a variety of cameras, operating in a range of clinics (rural/urban, ophthalmologist-led/optometrist-led), we believe that it will be a suitable addition to a public diabetic screening program.

## Supplementary information

Supplementary Tables

Supplementary Figure 1

Supplementary Figure 2

## Data Availability

The data was collected prospectively from consented participants [ANZCTR - ACTRN12620000488909 - Universal Trial Number (UTN) U1111-1249-7630.], specifically for this study and can not be released to external bodies without their consent.
