# Supplementary material for: A multi-centre prospective evaluation of THEIA™ to detect diabetic retinopathy (DR) and diabetic macular oedema (DMO) in the New Zealand screening program
Source: Eye (Lond). 2022 Sep 3;37(8):1683–9. doi: 10.1038/s41433-022-02217-w (PMC10219993; doi:10.1038/s41433-022-02217-w)
Supplement: Supplementary file 1 — Supplementary Tables [file 41433_2022_2217_MOESM1_ESM.docx]

*Supplementary Table 1: Eye-level (Overall ref/non-ref) THEIA™ performance vs gold standard*

| Retinopathy | Accuracy | Confusion matrix | Specificity | Sensitivity |
| --- | --- | --- | --- | --- |
| Auckland Central DHB (DHB) | 98.3% | [1220 23]  [ 4 323] | 99.7%  93.4% | 98.1%  98.8% |
| Optometrist led | 100.0% | [92 0]  [ 0 40] | 100.0%  100.0% | 100.0%  100.0% |
| Overall | 98.4% | [1312 23]  [ 4 363] | 99.7%  94.0% | 98.3%  98.9% |

*Supplementary Table 2: Eye-level (Retinopathy ref/non-ref) THEIA™ performance vs gold standard*

| Retinopathy | Accuracy | Confusion matrix | Specificity | Sensitivity |
| --- | --- | --- | --- | --- |
| Auckland Central DHB (DHB) | 98.3% | [1301 23]  [ 4 242] | 99.7%  91.3% | 98.3%  98.4% |
| Optometrist led | 100.0% | [96 0]  [ 0 36] | 100.0%  100.0% | 100.0%  100.0% |
| Overall | 98.4% | [1397 23]  [ 4 278] | 99.7%  92.4% | 98.4%  98.6% |

*Supplementary Table 3: Eye-level (Maculopathy ref/non-ref) THEIA™ performance vs gold standard*

| Maculopathy | Accuracy | Confusion matrix | Specificity | Sensitivity |
| --- | --- | --- | --- | --- |
| Auckland Central DHB (DHB) | 97.5% | [1311 30]  [ 10 219] | 99.2%  88.0% | 97.8%  95.6% |
| Optometrist led | 97.7% | [111 0]  [ 3 18] | 97.4%  100.0% | 100.0%  85.7% |
| Overall | 97.5% | [1422 30]  [ 13 237] | 99.1%  88.8% | 97.9%  94.8% |

*Supplementary Table 4: Eye-level retinopathy grade results*

| 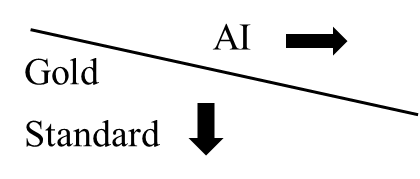 | None Detected | Mild | mtmDR | sight threatening |
| --- | --- | --- | --- | --- |
| None Detected | 873 | 151 | 0 | 0 |
| Mild | 52 | 321 | 22 | 1 |
| mtmDR | 0 | 4 | 46 | 84 |
| Sight threatening | 0 | 0 | 1 | 147 |

*Supplementary Table 5: Eye-level maculopathy grade results*

| 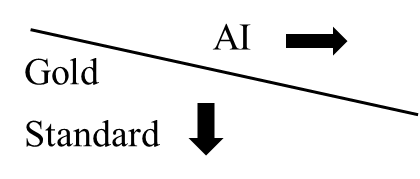 | None Detected | Mild | mtmDR | sight threatening |
| --- | --- | --- | --- | --- |
| None Detected | 1089 | 41 | 1 | 6 |
| Mild | 59 | 233 | 5 | 18 |
| mtmDR | 1 | 10 | 35 | 28 |
| sight threatening | 0 | 2 | 5 | 169 |

*Supplementary Table 6: Eye-level combined retinopathy & maculopathy grade results*

| 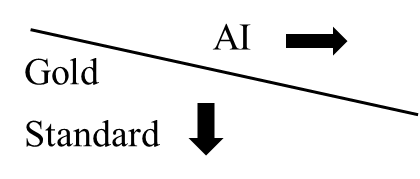 | None Detected | Mild | mtmDR | sight threatening |
| --- | --- | --- | --- | --- |
| None Detected | 868 | 217 | 2 | 6 |
| Mild | 28 | 207 | 16 | 60 |
| mtmDR | 0 | 3 | 27 | 62 |
| sight threatening | 0 | 0 | 1 | 216 |

*Supplementary Table 7: Patient-level THEIA™ confusion matrix using the None Detected, Mild, mtmDR, Sight threatening, grading scheme for individuals recruited in the Optometrist led practice. (Eidon camera)*

| 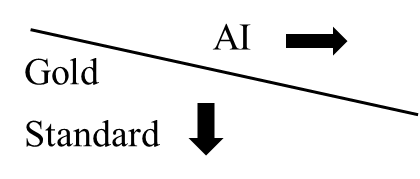 | None Detected | Mild | mtmDR | SIGHT threatening |
| --- | --- | --- | --- | --- |
| None Detected | 21 | 10 | 0 | 0 |
| Mild | 5 | 13 | 0 | 0 |
| mtmDR | 0 | 0 | 4 | 3 |
| SIGHT threatening | 0 | 0 | 1 | 30 |

*Supplementary Table 8: Patient-level THEIA™ confusion matrix using the None Detected, Mild, mtmDR, Sight threatening grading scheme for individuals recruited in the hospital led program. (Canon cameras)*

| 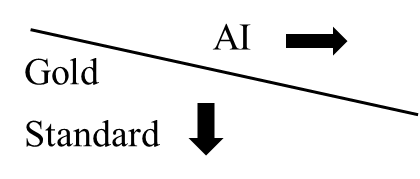 | None Detected | Mild | mtmDR | SIGHT threatening |
| --- | --- | --- | --- | --- |
| None Detected | 320 | 113 | 0 | 1 |
| Mild | 17 | 147 | 2 | 8 |
| mtmDR | 0 | 0 | 13 | 27 |
| SIGHT threatening | 0 | 0 | 0 | 165 |

*Supplementary Table 9: Audit of discordant grades issued*

|  | FINAL adjudicated. | | | | THEIA predicted | | | | Discrepancy | Explanation |
| --- | --- | --- | --- | --- | --- | --- | --- | --- | --- | --- |
|  | Right eye | | Left eye | | Right eye | | Left eye | |  |  |
| Patient ID | R Grade | M Grade | R Grade | M Grade | R Grade | M Grade | R Grade | M Grade |  |  |
| TOKU-1030 | 2 | 1 |  |  | 3 | 1 |  |  | RE: R3 issued but R2 | R Just below threshold for R3. |
| TOKU-1072 | 1 | 2 |  |  | 0 | 4 |  |  | RE: M4 issued but M2 | Thrombosed MA |
| TOKU-1084 | 1 | 2 | 1 | 2 | 1 | 2 | 1 | 4 | LE: M4 issued but M2 | Hard drusen |
| TOKU-1120 | 0 | 0 | 0 | 0 | 0 | 0 | 0 | 4 | LE: M4 issued but M2 | Thrombosed MA |
| TOKU-1202 | 0 | 0 | 1 | 2 | 1 | 4 | 1 | 2 | RE: R1 issued but R0. LE:M4 issued but M0 | Thrombosed MA |
| TOKU-1258 | 0 | 0 | 1 | 0 | 1 | 4 | 3 | 0 | RE: M4 issued but M0. LE R3 issued but R1 | RE Pachy drusen mimicking exudate. LE: 2 blots left eye only |
| TOKU-1573 | 1 | 2 | 1 | 2 | 1 | 2 | 1 | 4 | LE: M4 issued but M0 | LE: Pachydrusen mimicking exudate |
| TOKU-1593 | 3 | 2 | 2 | 2 | 3 | 2 | 2 | 4 | LE: M4 issued but M2 | RE Hard drusen. |
| TOKU-1627 | 1 | 2 |  |  | 1 | 4 |  |  | RE: M4 issued but M2 | Linear Hard drusen |
| TOKU-1665 | 1 | 0 | 1 | 0 | 0 | 0 | 1 | 4 | LE: M4 issued but M0 | Linear Hard drusen |
| TOKU-1784 | 1 | 1 | 1 | 1 | 3 | 1 | 1 | 1 | RE: R3 issued but R1 | RE: Just below threshold R3. |

*Supplementary Table 10: Demographics of the participants recruited in this study*

| Characteristics | | Optometry led | Ophthalmologist led | Overall |
| --- | --- | --- | --- | --- |
| Age (years) | Mean | 64 | 60.2 | 60.6 |
|  | Median | 64.7 | 61 | 61.4 |
|  | Range | 69 | 70 | 70 |
| Ethnicity | African | 7 | 17 | 3% |
|  | White | 58 | 242 | 33% |
|  | Asian | 2 | 214 | 24% |
|  | Polynesian | 7 | 251 | 29% |
|  | Other | 13 | 89 | 11% |
| Gender | Female (%) | 47 | 381 | 48% |
| Total |  | 87 | 813 | 900 |
