# Supplementary figures and images for: A multi-centre prospective evaluation of THEIA™ to detect diabetic retinopathy (DR) and diabetic macular oedema (DMO) in the New Zealand screening program
Source: Eye (Lond). 2022 Sep 3;37(8):1683–9. doi: 10.1038/s41433-022-02217-w (PMC10219993; doi:10.1038/s41433-022-02217-w)

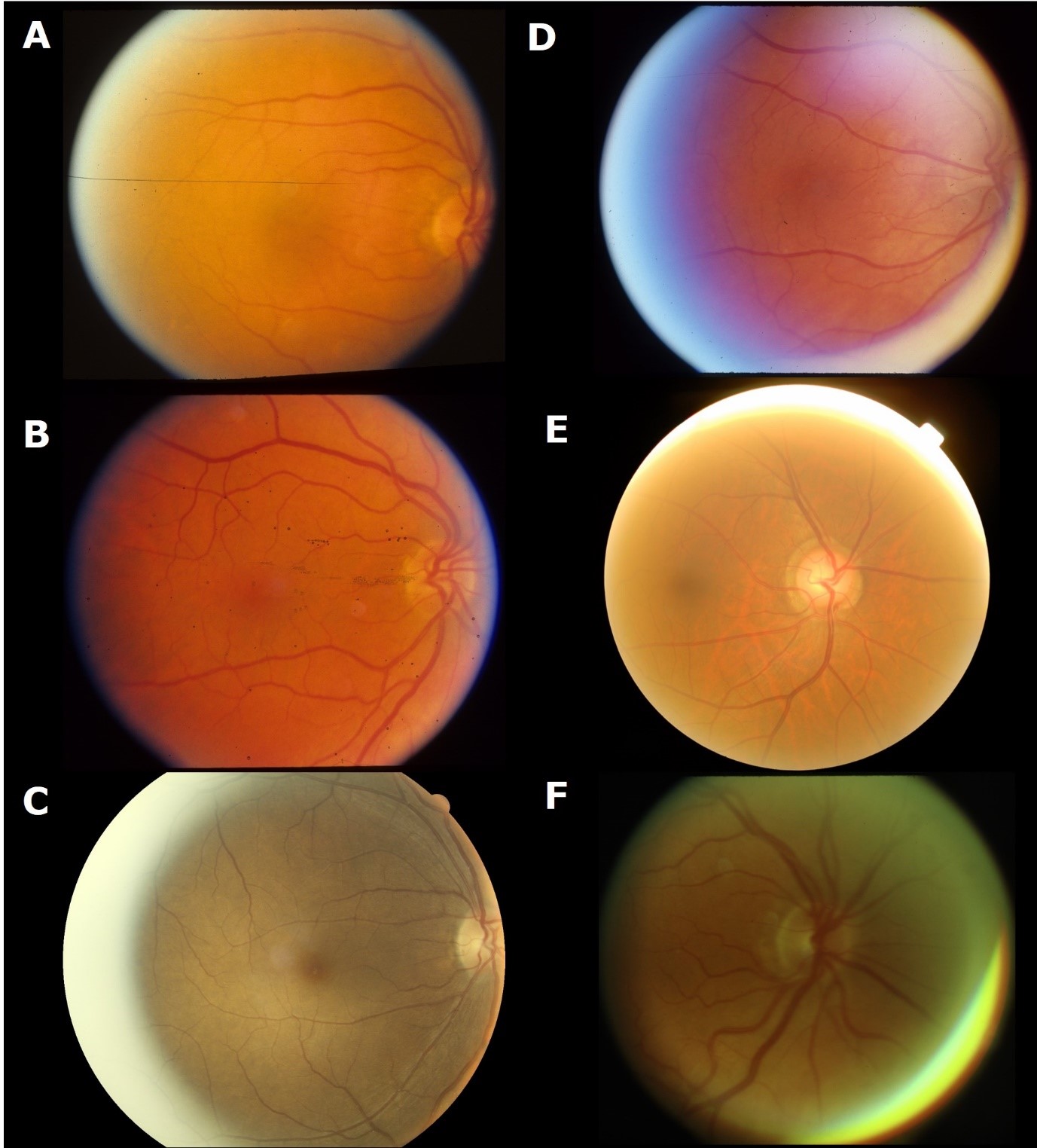

Supplement: Supplementary file 2 — Supplementary Figure 1 [file 41433_2022_2217_MOESM2_ESM.jpg]

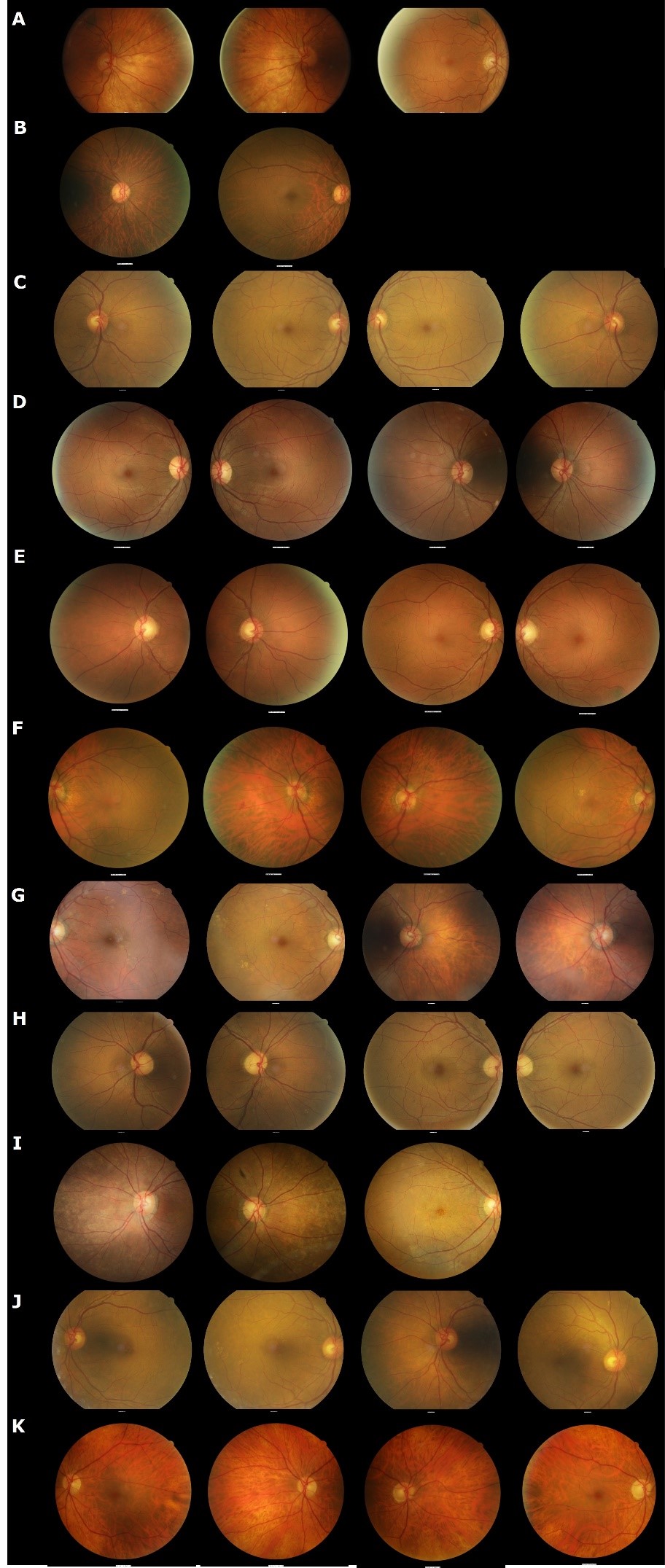

Supplement: Supplementary file 3 — Supplementary Figure 2 [file 41433_2022_2217_MOESM3_ESM.jpg]
